# Supplementary material for: Brain Magnetic Resonance Findings in 117 Children with Autism Spectrum Disorder under 5 Years Old
Source: Brain Sci. 2020 Oct 16;10(10):741. doi: 10.3390/brainsci10100741 (PMC7602717; doi:10.3390/brainsci10100741)
Supplement: Supplementary file 1 [file brainsci-10-00741-s001.pdf]

Supplementary Files

Table S1. Frequency of positive MRI reports in the three categories of ASD symptoms' severity level.

	All ASD children (*N* = 117)			
	**Negative MRI report (*N* = 53)**	**Positive MRI report (*N* = 64)**	***Statistical comparison***	***P***
Mild	3	10	Mild vs Moderate	0.203
Moderate	22	24	Moderate vs Severe	1
Severe	28	30	Severe vs Mild	0.128

Table S1 legend: Table S1 reports the statistical comparison of the frequency of children with a positive MRI report among the three (mild, moderate and severe) categories of ASD symptoms' severity level. Statistics: two-tailed Fisher exact test; Significance level < 0.05. Abbreviations: Mild, moderate, severe refer to the classification of ADOS-2's comparative scores in three levels of symptoms' severity.

Table S2. Prevalence of EEG abnormalities in children with ASD with and without positive MRI.

	**Children with ASD *n* = 117**	**High-functioning ASD *n* = 41**	**Developmental Delay-ASD *n* = 76**	**Statistics**
Available EEG reports	100	37	63	
Abnormal EEG	28 (15)	10 (9)	18 (7)	*P* = 1 (*P*= 0.373)
Aspecific abnomalities	14 (9)	5 (4)	9 (5)	*P* = .335 (*P* = 1)
Paroxysmal abnomalities	14 (7)	5 (5)	9 (2)	*P* = .335 (*P* = 0.422)
Epilepsy	4 (1)	2 (1)	2 (0)	*P* = 1 (*P* = 1)

Abbreviations: Brackets, *n* abnormal EEG associated with positive MRI; ASD, autism spectrum disorder; Statistics: two-tailed Fisher's exact test comparing HF-ASD and DD-ASD.

Supplementary Methods

To measure the developmental age, 14 children were administered the Griffiths Mental Development Scales [1] while the Psychoeducational Profile-revised [2] was used for 15 children. The Stanford-Binet Scale [3] was used for one patient. Nonverbal IQ was assessed using the Leiter International Performance Scale-Revised [4] in 38 children and the Wechsler Scales for 4 children: Preschool and Primary Scale of Intelligence, third edition [5] for one patient and Wechsler Intelligence Scales for Children [6] for 3 patients. Forty-five children were not able to complete any of the aforementioned tests due to lack of cooperation or anxiety. In those cases, clinical judgment of level of functioning was made by the child neuropsychiatrists.

Because of a lack of compliance of some of our young patients, neurocognitive testing was done on average 16 months after the MRI assessment (between 31 months before and 106 months after the MRI examination).

The reported data on cognitive level (Table 1) were therefore obtained using different tests which allowed us to assess the presence either of a developmental delay (DD) in children up to 4.11 years old or of an intellectual disability (ID) in children of 5.0 years old or older.

The cut-off for DD/ID was set at a non-verbal IQ/GD score =/< 70 (i.e., 2 SD or more below population mean) which constitutes the most commonly used cut-off in both clinical practice and research (see for example [7–11]). For the sake of simplicity, we decided to label all children with ASD presenting with a non-verbal IQ/GD </= 70 as ASD-DD.

A proportion of 16.24% (19/117) of the children were younger than 31 months at the time of scanning (a final diagnosis of ASD was confirmed when they reached at least 31 months), 8 of them were assigned to a HF category, while the remaining 11 children have been considered as ASD-DD. The remaining 83.76% (98/117) were above 31 months at the time of the MRI assessment (65 ASD-DD and 33 ASD-HF).

Concerning the distribution frequency across the IQ ranges of the ASD-HF children, 48,78% (20/41) children fell in the 71-85 GD/IQ range, 24.39% (10/41) in the 86-100 GD/IQ range, 12.20% (5/41) in the 101-115 GD/IQ range and 14.63% (6/41) in the 116-130 GD/IQ range.

One might question whether the temporal misalignment between neurocognitive and MRI assessments could have affected the attribution of the children to one or another ASD category (namely, ASD-HF or ASD-DD). While for the children that underwent neurocognitive testing prior to MRI, it was not always possible to access successive neurocognitive scores, if present, in order to confirm our ASD-HF/DD classification, strong evidence has shown that childhood IQ appears to remain stable over time [12]. In all cases, for which a follow up of Developmental level/IQ was available, Child Neuropsychiatrists found a stability over time.

Supplementary References

1. Griffiths, R. *The Abilities of Young Children. Bucks: A Comprehensive System of Measurement for the First Eight Years of Life*. The Test Agency, Thames; Association for Research in Infant and Child Development: UK, **1984**; pp. 101–172.
2. Schopler, E. *Individualized Assessment and Treatment for Autistic and Developmentally Disabled Children: Psychoeducational Profile Revised (Pep-R)*; Pro-Ed: Austin, TX, USA, **1990**.
3. Terman, L.M.; Merrill, M.A. *Stanford-Binet Intelligence Scale: Manual for the Third Revision, Form L-M.*; Houghton Mifflin: Dublin, Ireland, **1960**.
4. Roid, G.M.; Miller, L.J. *Leiter International Performance Scale-Revised: Examiners Manual*; Stoelting Co.: Wood Dale, IL, USA, **1997**.
5. Wechsler, D. *The Wechsler Preschool and Primary Scale of Intelligence,* 3rd ed. (WPPSI-Ill); The Psychological Corporation: San Antonio, TX, USA, **2002**.
6. Wechsler, D. *The Wechsler Intelligence Scale for Children*, 4th ed; Pearson: London, UK, **2003**.
7. Bartak, L.; Rutter, M. Differences between mentally retarded and normally intelligent autistic children. *J. Autism Child. Schizophr.* **1976**, *6*, 109–120, doi:10.1007/BF01538054.
8. De Giambattista, C.; Ventura, P.; Trerotoli, P.; Margari, M.; Palumbi, R.; Margari, L. Subtyping the Autism Spectrum Disorder: Comparison of Children with High Functioning Autism and Asperger Syndrome. *J. Autism Dev. Disord*. **2019**, *49*, 138–150, doi:10.1007/s10803-018-3689-4.
9. Oliveras-Rentas, R.E.; Kenworthy, L.; Roberson, R.B.; Martin, A.; Wallace, G.L. WISC-IV profile in high-functioning autism spectrum disorders: Impaired processing speed is associated with increased autism communication symptoms and decreased adaptive communication abilities. *J. Autism Dev. Disord.* **2012**, *42*, 655–664, doi:10.1007/s10803-011-1289-7.
10. Szatmari, P. The Classification of Autism, Asperger's Syndrome, and Pervasive Developmental Disorder. *Can. J. Psychiatry* **2000**, *45*, 731–738, doi:10.1177/070674370004500806.
11. Cai, J.; Hu, X.; Guo, K.; Yang, P.; Situ, M.; Huang, Y. Increased Left Inferior Temporal Gyrus Was Found in Both Low Function Autism and High Function Autism. *Front. Psychiatry* **2018**, doi:10.3389/fpsyt.2018.00542, PMID: 30425664, PMCID: PMC6218606.
12. Magiati, I.; Tay, X.W.; Howlin, P. Cognitive, language, social and behavioural outcomes in adults with autism spectrum disorders: A systematic review of longitudinal follow-up studies in adulthood. *Clin. Psychol. Rev.* **2014**, *34*, 73–86, doi:10.1016/j.cpr.2013.11.002.
